# Quantifying Influencing Factors of Dioxin Removal in Fly Ash Pyrolysis Through Meta-Analysis and Structural Equation Modeling

**DOI:** 10.3390/toxics13121072

**Published:** 2025-12-12

**Authors:** Tao He, Shihan Tan, Qi Su, Feifei Chen, Chenlei Xie, Yuchi Zhong, Shuai Zhang, Jiafeng Ding

**Affiliations:** 1 School of Engineering, Hangzhou Normal University, Hangzhou 310018, China; 2 Zhejiang Provincial Key Laboratory of Wetland Intelligent Monitoring and Ecological Restoration, Hangzhou 311121, China; 3 Eco-Environmental Science Research and Design Institute of Zhejiang Province, Hangzhou 311121, China; 4 Hangzhou Fuyang Huilong Environmental Protection Technology Co., Ltd., Hangzhou 330183, China

**Keywords:** dioxins, fly ash, meta-analysis, removal efficiency, SEM

## Abstract

The treatment of polychlorinated dibenzodioxins and polychlorinated dibenzofurans (PCDD/Fs) in incineration fly ash presents a significant challenge in solid hazardous waste management. This study systematically analyzed the influence mechanisms of multiple factors on the removal efficiency of PCDD/Fs during fly ash pyrolysis. It integrated 4068 datasets conducted between 2010 and 2025 through meta-analysis. Results show that Al_2_O_3_, CaO, SiO_2_, and Cl in fly ash components enhance the removal efficiency by 14.0%, while Fe_2_O_3_ (Content greater than 5.7%) exhibits inhibitory effects. Cd and Cr demonstrate a bimodal response pattern: low/high concentrations promote removal, while medium concentrations inhibit it. Process optimization identified the optimal parameter combination as pyrolysis temperatures of 500–900 °C, residence time of 50–90 min, and a gas flow rate greater than or equal to 400 mL/min. A significant negative correlation was observed between the initial dioxin concentration and removal efficiency. This study established a structural equation modeling (SEM) model to describe how metallic and nonmetallic components, fly ash components, and pyrolysis conditions determine removal efficiency. Fly ash composition was confirmed as the most influential factor (total effect = 0.3194), with fixed carbon and ash content being the most reliable indicators. Among pyrolysis conditions, gas conditions (flow rate, gas type) also significantly affected removal efficiency (total effect = 0.2357). Conversely, nonmetallic components and excessively prolonged pyrolysis time (beyond the window) consistently reduced removal efficiency. These findings provide theoretical support for upgrading fly ash pyrolysis processes toward low-carbon and resource-efficient operations.

## 1. Introduction

With the rapid advancement of urbanization and economic development, the global annual production of municipal solid waste (MSW) has reached approximately 2.01 billion tons, projected to increase to 4 billion tons by 2026 [1]. Among solid waste disposal technologies, sanitary landfilling faces significant criticism due to its high environmental risks, while MSW incineration (MSWI) has gradually become mainstream for its ability to reduce secondary pollution and enable power generation and heat recovery [2,3]. Approximately 78.5% of Japan’s MSW is treated through incineration, while China’s MSW incineration rate has surged from 35.0% in 2014 to 79.9% in 2022 [4]. Fly ash generated from waste incineration contains toxic heavy metals and persistent organic pollutants such as dioxins, classifying it as hazardous waste. Conducting systematic environmental risk assessments and developing efficient technologies for harmless disposal and resource recovery are urgent priorities [5,6,7,8].

Currently, fly ash treatment methods include physical, chemical, and biological approaches [9]. Among physical methods, screening separates particles based on size but struggles to completely eliminate minute harmful substances; magnetic separation utilizes magnetic materials to remove ferromagnetic impurities, yet its effectiveness in removing non-magnetic substances is limited. Chen et al. investigated the use of SiO_2_-Al composite additives in conjunction with ball milling to treat fly ash [10]. This process reduced the total toxicity equivalent concentration of PCDD/Fs from an initial 4.2 ng I-toxicity equivalent (TEQ)/kg to 0.8 ng I-TEQ/kg, achieving a degradation efficiency of 81.0%. Among chemical methods, chemical stabilization involves adding specific chemicals (e.g., phosphates) to form stable precipitates of heavy metals, reducing their reactivity and mobility. However, this approach may introduce new chemical substances causing secondary pollution, and precise control of chemical dosage is required [11,12]. For instance, molten salt thermal treatment has been proposed for treating pollutants in MSWI fly ash under relatively mild conditions (600–800 °C), achieving detoxification through removal or stabilization processes [13]. Biological methods leverage microbial degradation of pollutants, offering environmental advantages but characterized by extended treatment cycles, unstable efficiency, and susceptibility to environmental factors [14]. Studies indicate that bioleaching technology combined with electro-remediation has been applied to remove pollutants from MSW incineration fly ash. For instance, Hu et al. employed a two-step process using a bioelectrochemical system and an electrolytic reactor to remove pollutants from fly ash, achieving removal efficiencies exceeding 95% in both stages [15].

Among these treatment methods, pyrolysis has emerged as a promising technology. By subjecting fly ash to high-temperature heating in an oxygen-free or low-oxygen environment, it decomposes dioxins and certain inorganic components, converting them into low-molecular-weight gases or low-toxicity/non-toxic substances [16,17]. However, the fly ash pyrolysis process is complex, and the removal efficiency of dioxins is influenced by multiple factors, including pyrolysis conditions, fly ash properties, and the characteristics of the dioxins themselves. Pyrolysis conditions, such as temperature, show that a 100 °C increase can boost dioxin decomposition rates by approximately 20% [18]. Regarding sample quantity, excessive fly ash samples may lead to incomplete pyrolysis, impairing removal efficiency. Concerning fly ash properties, CaO exhibits significant catalytic activity in promoting the decomposition of dioxins. MSWI fly ash contains substantial PCDD/Fs, and CaO can facilitate the dichlorination and cracking reactions of these highly toxic organics by providing an alkaline environment. Experiments demonstrate that under low temperature pyrolysis conditions at 350 °C, adding CaO reduces total dioxin concentration from 9582.1 ng/kg to 43.8 ng/kg, achieving a TEQ reduction rate exceeding 98%. Regarding dioxin characteristics, PCDD/Fs with lower boiling point volatilize and decompose more readily during pyrolysis. At excessively high concentrations, pollutants may interact with each other, reducing pyrolytic removal efficiency. Research indicates that in certain systems, the dichlorination rate of highly chlorinated PCDD/Fs significantly accelerates, while the accumulation of low-chlorinated products is suppressed. This reduces the I-TEQ value from an initial 9.8 ng I-TEQ/g to below 0.2 ng I-TEQ/g, achieving a toxicity equivalent reduction rate exceeding 98.5% [19].

To comprehensively analyze these influencing factors, meta-analysis plays a crucial role. As an advanced statistical method, it quantitatively combines and analyzes results from multiple independent studies, addressing the limitations of small sample sizes and potential bias in individual research. In the field of fly ash pyrolysis research, meta-analysis can extract data from extensive literature on how various factors influence pyrolysis effectiveness. For instance, by collecting research data from different laboratories or actual engineering projects regarding the impact of pyrolysis conditions on dioxin removal efficiency, meta-analysis can comprehensively account for the differences among these studies. This approach yields the average dioxin removal efficiency across different conditions and establishes a quantitative relationship between factors and removal efficiency. Simultaneously, meta-analysis can assess heterogeneity among studies and analyze causes of result discrepancies (such as differences in experimental equipment or operating conditions), thereby providing more accurate and universally applicable references for subsequent research. This approach helps researchers gain a better understanding of complex environmental issues, offers theoretical support for developing effective treatment strategies, and establishes the data foundation and theoretical framework for constructing dioxin removal efficiency prediction models [20].

Stepwise regression is a statistical modeling method that iteratively selects variables to identify the subset most explanatory of the dependent variable from a large pool of candidate independent variables [21]. Its core principle involves sequentially adding or removing variables while using statistical criteria to assess their significance, ultimately constructing a model with optimal predictive power [22]. In fly ash pyrolysis studies, stepwise regression can determine the relative influence weights of each condition on removal efficiency. The model may prioritize retaining significant variables while eliminating non-significant factors. This approach balances model complexity and interpretability, laying groundwork for structural equation modeling (SEM) [23]. SEM is a multivariate statistical analysis method that integrates measurement and analysis. It can simultaneously handle multiple dependent variables, accommodate measurement errors in variables, and analyze direct and indirect relationships between variables [24]. SEM can be used to investigate the pathways through which factors influence pyrolysis outcomes in fly ash treatment. It can explore how pyrolysis conditions, fly ash properties, and other factors affect dioxin removal efficiency by influencing pyrolysis processes. This approach helps researchers gain a more in-depth understanding of complex environmental issues, provides theoretical support for developing effective treatment strategies, and offers methodological support for clarifying variable relationships [25].

This study employed a meta-analysis approach to systematically evaluate factors influencing dioxin removal efficiency through literature review and the construction of a multidimensional database. Standardized effect size analysis identified primary drivers, quantified their direction and magnitude of influence, and explored synergistic relationships among multiple factors. This approach helps overcome the limitations of single studies, such as restricted sample sizes and conclusions susceptible to specific conditions, providing more universally applicable data support for understanding the removal mechanisms of PCDD/Fs during fly ash pyrolysis. The findings offer references for optimizing pyrolysis process parameters and enhancing PCDD/Fs removal efficiency, further advancing the exploration of pyrolysis technology in the harmless treatment of fly ash.

## 2. Materials and Methods

### 2.1. Data Source

**Figure 1:**
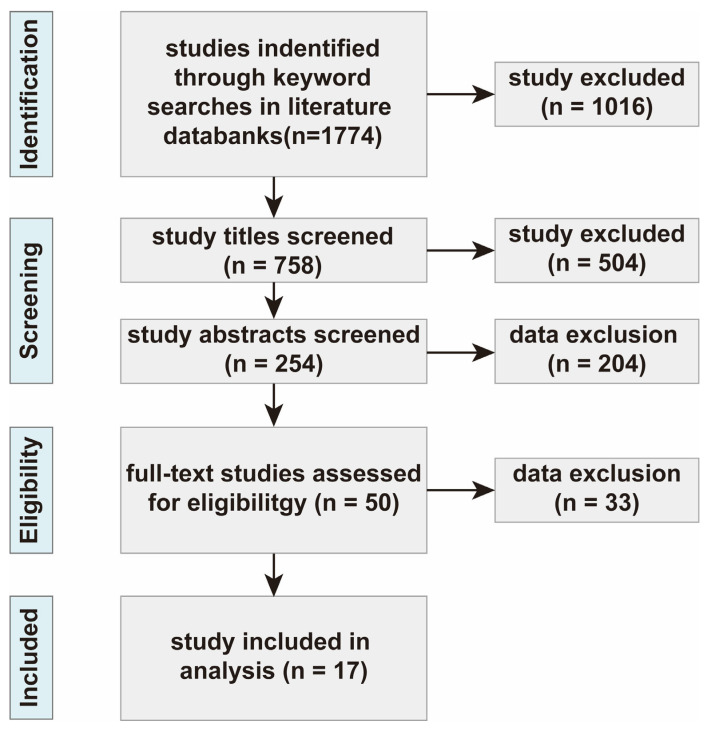
PRISMA flow diagram. “n” represents the number of papers relevant to the screening.

This study utilized two databases: Web of Science Core Collection and Google Scholar, targeting publications from January 2010 to June 2025. A comprehensive literature search was conducted using the keywords fly ash, pyrolysis, dioxins, and removal efficiency. The specific literature search criteria can be found in Text S1. The selection of literature adhered to the preferred reporting items for systematic reviews and meta-analyses (PRISMA) guidelines (Figure 1).

During the initial screening phase, duplicate records were removed using EndNote X9, and irrelevant studies were excluded based on titles and abstracts, such as those unrelated to pyrolysis processes, non-fly ash matrices, and review articles. Ultimately, 17 studies meeting the following criteria were selected for data extraction and inclusion in the meta-analysis: (1) randomized experimental design; (2) Specified fly ash treatment method as “pyrolysis”; (3) Organic pollutants removed were dioxin compounds; (4) Successful removal of at least one PCDD/Fs was evaluated; (5) All treated raw materials contained only fly ash as the contaminant, with no other pollutants; (6) All data and relevant information were obtainable from the articles or extractable from tables and figures.

### 2.2. Database Construction

Data was collected and entered into Microsoft Excel 2021 software. The complete information in the database is primarily divided into three major categories. The first category pertains to fly ash properties: weight (g), ash content (%), fixed carbon content (%), volatile matter content (%), Fe_2_O_3_ content (%), Al_2_O_3_ content (%), CaO content (%), SiO_2_ content (%), Cl content (%), Cd content (%), Cr content (%). The second category covers pyrolysis conditions: time (min), temperature (°C), gas type, and gas flow rate (mL/min); The third category is dioxin characteristics: boiling point (°C), melting point (°C), Henry’s constant (atm·m^3^/mol), Lg*K*_ow_, initial concentration (pg/g). Where data were reported in graphical formats, these values were indirectly extracted using GetData GRAPH Digitizer 2.24 software. Since the primary focus of this study is dioxins, and among dioxins, 2,3,7,8-Tetrachlorodibenzo-para-dioxin (2,3,7,8-TCDD) possesses the highest toxicity equivalent factor among dioxin compounds [26,27]. It has been widely established by the World Health Organization as the benchmark reference substance for toxicological assessment. Its structure provides comprehensive coverage, so data from 2,3,7,8-TCDD removal are designated as the control group, while the remaining data constitute the experimental group. To systematically analyze the effects of fly ash properties, pyrolysis conditions, and dioxin characteristics on the removal efficiency of PCDD/Fs in fly ash via pyrolysis, all data were grouped using standardized methods. Fly ash properties and pyrolysis conditions underwent three-group standardized processing. Given that dioxin characteristics possess inherent chemical attributes, this study focused on molecular mass as a key parameter for continuous variable analysis, avoiding attribute fragmentation caused by discrete grouping. Specific details are referenced.

### 2.3. Data Cleaning, Outlier Detection, and Sensitivity Analysis

To ensure the reliability and comparability of the extracted data, a multi-stage data-quality control procedure was implemented prior to meta-analysis. First, missing values in continuous covariates were imputed using the multiple imputation by chained equations algorithm, which preserves the multivariate structure of the dataset and prevents potential bias associated with listwise deletion. Second, extreme values were identified using the interquartile range (IQR) rule, where observations falling below Q1 − 1.5 × IQR or above Q3 + 1.5 × IQR were classified as outliers. This non-parametric criterion is widely used in environmental and materials science to detect anomalous measurements arising from inconsistent pyrolysis conditions. To evaluate the robustness of the synthesized effect size, a leave-one-study-out sensitivity analysis was performed within a multilevel random-effects framework. For each iteration, one study was removed, and the model was refitted to examine the extent to which the pooled estimate changed in response to study-level perturbation. Stability of the effect size across iterations was used as an indicator of low influence from individual studies, thereby confirming the robustness of the final meta-analytic conclusions.

### 2.4. Meta-Analysis

Meta-analysis, as a statistical method, quantitatively combines data from similar studies to enhance statistical power, investigate consistency and differences among studies, and provide reliable evidence for scientific decision-making [28]. R software (4.5.2) and Review Manager 5.4 were employed to calculate the effects of fly ash properties, pyrolysis conditions, and dioxin characteristics on the removal efficiency of PCDD/Fs during fly ash pyrolysis [29,30]. Each dataset (experimental and control groups) must include the mean, standard deviation, and sample size (Tables S1–S3) [31]. Response ratio (RR) is treated as the logarithmically transformed ratio of means for each pair of comparisons (Equation (1)):

(1) $$RR=\ln(X_{B}/X_{C})$$

where *X*_B_ and *X*_C_ represent the mean removal efficiencies of dioxin in fly ash during pyrolysis for the control group and experimental group. When *RR* equals 0, the condition has no effect on dioxin removal efficiency. When *RR* is greater than 0, it indicates that the condition has a positive effect on dioxin removal efficiency. When *RR* is less than 0, it indicates that the condition has a negative effect on dioxin removal efficiency.

Equation (2) is used to calculate the variance (*v*) of the *RR* for each study:

(2) $$v=\frac{S_{t}^{2}}{n_{t}\times X_{t}^{2}}+\frac{S_{t}^{2}}{n_{c}\times X_{c}^{2}}$$

where *S*_t_ and *S*_c_ represent the standard deviation of the control group and experimental group, respectively. *n*_t_ and *n*_c_ denote the sample sizes of the experimental group and control group, respectively [32].

The calculation of the weighting factor, weighted response ratio (*R*_+_), standard error of *R* (*S*), and 95% confidence interval (CI) for *R* is as follows (Equations (3)–(6)):

(3) $$w_{i}=\frac{1}{v}$$

(4) $$R_{+}=\frac{\sum_{i=1}^{m}\;\sum_{j=1}^{k}w_{ij}R_{ij}}{\sum_{i=1}^{m}\;\sum_{j=1}^{k}w_{ij}}$$

(5) $$S=\sqrt{\frac{1}{\sum_{i=1}^{m}\;\sum_{j=1}^{k}w_{ij}}}$$

(6) $$95\%CI=R_{+}\pm 1.96S$$

where *i* and *j* represent data points *i* and *j,* respectively. *m* and *k* denote the comparison values in the control and experimental groups, respectively. *w*_ij_*R*_ij_ and *w*_ij_ are the weighted response ratios and adjusted weighting factors of the observed values. When the 95% CI does not overlap with zero, the removal efficiency is considered significantly increased (>0) or decreased (<0) compared to the control group for these two variables (*p* < 0.05) [33].

The percentage change in pollutant removal efficiency response under a specific condition is calculated using formula (Equation (7)):

(7) $$Percentage(\%)=(e^{R+}-1)\times 100\%$$

Forest plots were used to visually summarize the effect sizes (weighted response ratio, RR) and their 95% confidence intervals (95% CI) across different studies or conditions. Each data point represents the estimated effect, with horizontal lines indicating the confidence interval; if the line does not cross zero, the effect is considered statistically significant (*p* < 0.05).

### 2.5. Stepwise Regression Model

Stepwise regression is a multivariate linear regression variable selection method based on statistical significance criteria. By iteratively introducing or removing independent variables, it ultimately constructs the regression model with the strongest explanatory power and simplest structure [34]. This method effectively balances model predictive accuracy and complexity, avoiding overfitting issues caused by excessive variables [35]. In this study, dioxin removal efficiency serves as the dependent variable, while fly ash properties, pyrolysis conditions, and dioxin characteristics function as candidate independent variables. Forward selection or backward elimination is performed sequentially until the model achieves an optimal fit. The significant variables retained after screening are incorporated as latent variables into subsequent modeling, thereby enhancing the model’s robustness and interpretability.

### 2.6. Structural Equation Modeling

SEM is a multivariate statistical analysis method based on the covariance matrix of variables. It integrates measurement models and structural models to comprehensively examine the complex relationships between observed variables (manifest variables) and latent constructs (latent variables) [36]. Measurement models characterize how manifest variables reflect latent variables, validating their reliability and validity. SEM further reveals direct and indirect causal pathways among latent variables, making them suitable for exploring multilevel, multidimensional systemic mechanisms. In this study, SEM was employed to analyze the intrinsic network of relationships among each factor during fly ash pyrolysis. Compared to traditional regression methods, SEM accommodates measurement errors in variables and supports simultaneous analysis of multiple dependent variables. This approach not only enhances model robustness but also enables the separation and identification of direct and indirect effects between variables, providing robust statistical support for revealing multi-factor synergistic regulatory mechanisms [37].

### 2.7. Statistical Analysis

Statistical significance was assessed using one-way analysis of variance (ANOVA) followed by Tukey’s multiple comparison tests. Review Manager 5.4 software analyzed each dataset under every condition, yielding weighted effect sizes and 95% CI. All figures were generated using R (version 4.5.2), Origin 2021, and Adobe Illustrator 2023.

## 3. Results and Discussion

### 3.1. Dataset Analysis

This comprehensive meta-analysis encompassed 17 studies, involving 17 different fly ash samples and 118 PCDD/Fs (Table S4). The analysis focused on examining the specific roles of 20 variables in determining the removal efficiency of PCDD/Fs during fly ash pyrolysis. A multi-stage data cleaning procedure was adopted to ensure data quality and the suitability of the dataset for meta-analysis. Ultimately, a total of 4068 data entries were extracted. Among these 20 variables, gas type was categorized as a characteristic variable. To facilitate analysis, gas type was converted into a numerical variable by indexing it based on the relative molecular mass of the carrier gas. The specific conversion method is detailed in Table S5.

**Figure 2:**
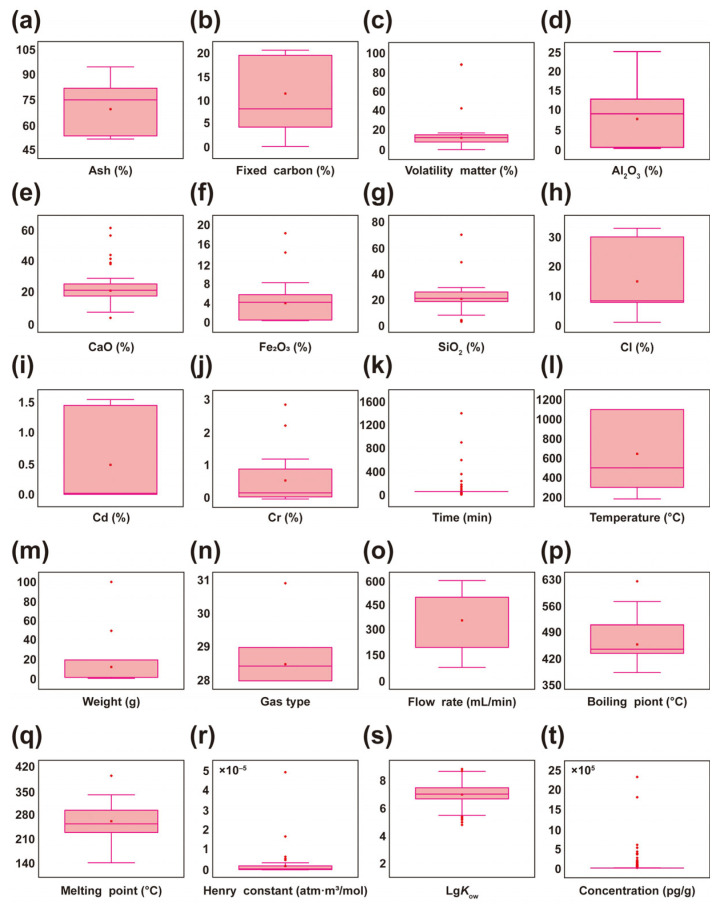
Box plots for each characteristic. (**a**) Ash; (**b**) Fixed carbon; (**c**) Volatile matter; (**d**) Al_2_O_3_; (**e**) CaO; (**f**) Fe_2_O_3_; (**g**) SiO_2_; (**h**) Cl; (**i**) Cd; (**j**) Cr; (**k**) Time; (**l**) Temperature; (**m**) Weight; (**n**) Gas type; (**o**) Flow rate; (**p**) Boiling point; (**q**) Melting point; (**r**) Henry constant; (**s**) Lg*K*_ow_; (**t**) Concentration.

The distribution of box plots for 20 variables indicates that significant differences exist in the distribution of ash (Figure 2a), fixed carbon (Figure 2b), volatile matter content (Figure 2c), and the content of various oxides and elements in fly ash. Specifically, ash and fixed carbon content showed significant variation across different samples, while volatile matter content exhibited relatively consistent distribution. Regarding oxides and elemental content, Al_2_O_3_ (Figure 2d) and CaO (Figure 2e) displayed scattered distributions, whereas Fe_2_O_3_ (Figure 2f) and SiO_2_ (Figure 2g) data were relatively concentrated. For oxides like Al_2_O_3_ and Fe_2_O_3_, the median values were centrally located, with most samples fluctuating around these values, though some exhibited substantial variation. Cl (Figure 2h) showed significant variation and scattered distribution across different fly ashes. Cd (Figure 2i) generally exhibited low and stable concentrations, with most data points clustered closely around the median. Cr (Figure 2j) data were concentrated but contained large outliers, indicating significant concentration fluctuations. Regarding pyrolysis conditions, time (Figure 2k) and temperature (Figure 2l) data exhibited broad overall ranges, indicating comprehensive pyrolysis coverage. Weight (Figure 2m) data showed relative concentration with some outliers. Gas type (Figure 2n) and flow rate (Figure 2o) data were dispersed, with flow rate outliers concentrated toward higher values, suggesting prominent high-flow conditions in experiments. Regarding PCDD/Fs properties, most boiling point (Figure 2p) and melting point (Figure 2q) cluster within lower ranges, indicating substantial variation potential. The median values for Henry’s constants (Figure 2r) and Lg*K*_ow_ (Figure 2s) are positioned in the middle of the box plot, with data showing a tendency to cluster around these values. The initial concentration (Figure 2t) exhibits high dispersion, primarily due to variations in fly ash properties across different experiments, directly influencing their initial concentrations.

A leave-one-study-out sensitivity analysis was performed using the multilevel random-effects model. The pooled effect estimates obtained after sequentially removing each study ranged from 89.39 to 90.20, corresponding to a fluctuation of only 0.9% relative to the full-model estimate (Table S6). This minimal variation indicates that no single study exerted a disproportionate influence on the overall effect size. Therefore, the meta-analytic results are considered highly robust and stable with respect to study-level perturbations.

### 3.2. Determinants of PCDD/Fs Removal Efficiency

#### 3.2.1. Fly Ash Properties Effects

**Figure 3:**
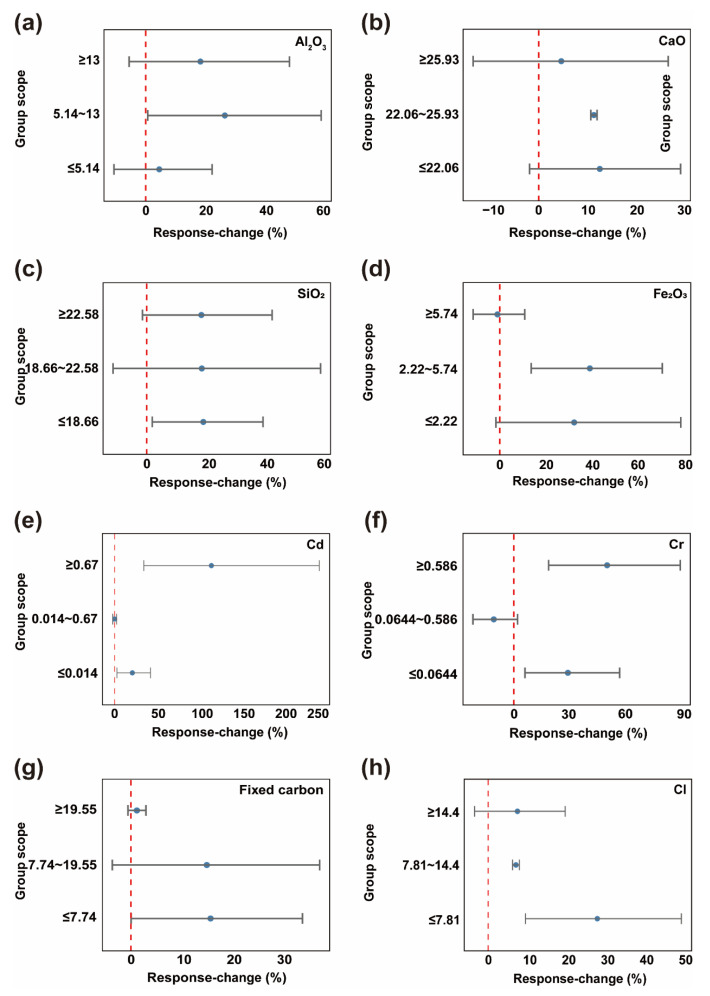
Forest plot of fly ash properties. Blue circles represent the point estimates of the weighted mean *RR* for each subgroup, with their horizontal position indicating the direction and magnitude of the effect. Gray horizontal lines denote the 95% Cl for this effect size, reflecting the precision of the estimate. Red dashed vertical lines mark the null line (*RR* = 0). (**a**) Al_2_O_3_; (**b**) CaO; (**c**) SiO_2_; (**d**) Fe_2_O_3_; (**e**) Cd; (**f**) Cr; (**g**) Fixed carbon; (**h**) Cl.

This section examines the factors influencing the removal efficiency of PCDD/Fs during fly ash pyrolysis. Specifically, metal oxides such as Al_2_O_3_ (Figure 3a), CaO (Figure 3b), SiO_2_ (Figure 3c), and Fe_2_O_3_ (Figure 3d) show a pronounced positive correlation with removal efficiency. This is primarily attributed to the unique catalytic activity and thermal conductivity exhibited by these compounds within the pyrolysis reaction system. Metal oxides provide abundant active sites for the pyrolysis reaction of PCDD/Fs, effectively lowering the activation energy of the reaction and thereby accelerating the pyrolysis process. They also promote uniform heat distribution within the system, further facilitating the deep removal of PCDD/Fs [38]. Notably, significant synergistic catalytic effects exist among these metal oxides. For instance, the Al_2_O_3_-and Fe_2_O_3_ composite system enhances dichlorination efficiency by promoting multistep reactions (such as adsorption-oxidation coupling mechanisms), thereby further improving PCDD/Fs removal. However, a peculiarity exists in the mechanism of Fe_2_O_3_: when its content reaches or exceeds 5.7%, PCDD/Fs removal efficiency paradoxically decreases compared to the control group. This anomaly can be attributed to the alteration of the reaction system’s physical structure caused by high Fe_2_O_3_ content. Excessive Fe_2_O_3_ may accumulate or agglomerate within the system, forming physical barriers that hinder effective contact between pollutant molecules and active sites. This impedes pollutant diffusion and reaction progression, ultimately negatively impacting removal efficiency. Its mechanism of action is relatively stable and less susceptible to interference from other factors, providing an important theoretical basis for optimizing pyrolysis conditions. It should be noted that fly ash often contains a substantial amorphous fraction that is not consistently quantified in the source literature. Since the compositions in our database are mainly reported as bulk oxide equivalents, the observed “oxide effects” should be interpreted as apparent overall associations. Unrecognized amorphous phases may modify the availability of active sites and the accessibility of chlorine and catalytic metals, potentially biasing or diluting oxide-specific catalytic interpretations and contributing to heterogeneity across studies.

For Cd (Figure 3e) and Cr (Figure 3f), the influence of their concentration exhibits a complex bimodal pattern: when the concentrations of these two elements are either low or high, they both show a positive promotion effect on PCDD/Fs removal efficiency. However, when Cd concentrations ranged from 0.014% to 0.67% and Cr concentrations ranged from 0.0644% to 0.586%, a significant negative impact was observed relative to the control group. This phenomenon stems from the chemical properties of Cd and Cr (such as their ability to gain or lose electrons in reactions), their catalytic effects (whether they promote or inhibit pyrolysis-related reactions), competitive adsorption behavior (competing with other substances for adsorption on active sites), redox properties (tendency to undergo oxidation or reduction in pyrolysis systems), chemical speciation (form of compound present), and physical properties (factors affecting contact and reaction with pollutants). This reflects the complex behavior patterns of heavy metals in pyrolysis reactions [39,40,41]. The influence of fixed carbon content (Figure 3g) on PCDD/Fs removal efficiency follows specific patterns: when the fixed carbon proportion is below 19.6%, it enhances PCDD/Fs removal, though the wide confidence interval span between the experimental and control groups indicates some uncertainty. However, when the fixed carbon proportion reached or exceeded 19.6%, the PCDD/Fs removal efficiency relative to the control group stabilized at approximately 1%. This result indicates that at high content levels, fixed carbon plays a significant role and may become one of the key factors influencing removal efficiency. It is clearly demonstrated that Cl (Figure 3h) enhances the removal efficiency of PCDD/Fs regardless of its proportion in fly ash. Particularly within the Cl content range of 7.8% to 14.4%, this enhancement effect exhibits high stability-the experimental group consistently maintained an efficiency improvement of approximately 14% compared to the control group. This stable effect arises from the combined regulation of multiple factors, including the high chemical reactivity of Cl itself and its synergistic interactions with other reactants [42,43].

#### 3.2.2. Pyrolysis Condition Impacts

**Figure 4:**
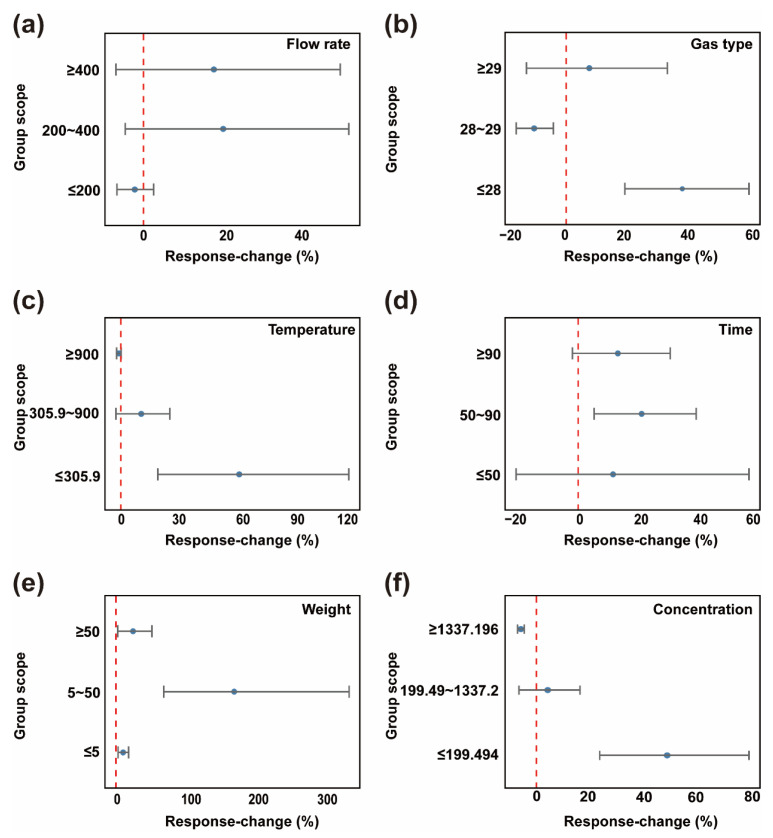
Pyrolysis conditions forest plot. Blue circles represent the point estimates of the weighted mean *RR* for each subgroup, with their horizontal position indicating the direction and magnitude of the effect. Gray horizontal lines denote the 95% Cl for this effect size, reflecting the precision of the estimate. Red dashed vertical lines mark the null line (*RR* = 0). (**a**) Flow rate; (**b**) Gas type; (**c**) Temperature; (**d**) Time; (**e**) Weight; (**f**) Concentration.

Pyrolysis conditions serve as critical indicators, playing a decisive role in the removal of PCDD/Fs from fly ash. Higher flow rates, specific gas types, appropriate high temperatures, extended treatment durations, moderate initial concentrations, and suitable weight typically yield higher removal efficiency responses. As shown in Figure 4a, gas flow rate significantly impacts removal efficiency. When the flow rate is greater than or equal to 400 mL/min, the percentage change in PCDD/Fs removal efficiency is relatively great, with removal efficiency increasing by approximately 20% compared to the control group. The 200–400 mL/min range showed a secondary effect. Conversely, when the gas flow rate is greater than or equal to 200 mL/min, PCDD/Fs removal efficiency decreased by approximately 5% compared to the control group. This may be attributed to a higher flow rate, accelerating weight transport and mixing, enabling more thorough pyrolysis reactions and thereby enhancing PCDD/Fs removal efficiency. Conversely, lower gas flow rates result in insufficient weight and heat transfer, preventing complete pyrolysis of PCDD/Fs and negatively impacting removal efficiency [44]. Regarding gas types, Figure 4b shows that when the relative molecular mass is less than or equal to 28 or greater than or equal to 29, PCDD/Fs removal efficiency increased by approximately 10% and 38%, respectively. Conversely, when the gas relative molecular mass was between 28 and 29, PCDD/Fs removal efficiency decreased by about 10%. This may result from competitive adsorption by gas molecules. These molecules may compete with PCDD/Fs for adsorption sites on fly ash surfaces. By preferentially adsorbing onto the fly ash surface, they hinder contact between PCDD/Fs and the active sites on the fly ash surface, making effective pyrolysis of PCDD/Fs difficult and reducing removal efficiency [45]. The effect of temperature (Figure 4c) on PCDD/Fs removal efficiency exhibits a phased characteristic: when the temperature is below 900.0 °C, it has a positive impact on removal efficiency. Specifically, at temperatures less than or equal to 305.9 °C, and within the 305.9–900.0 °C range, removal efficiency increased by approximately 60% and 10%, respectively. Within the range of 305.9 °C or below, the confidence interval is relatively wide, indicating that removal efficiency is highly sensitive to temperature changes. It should be noted that the temperature interval of approximately 250–400 °C is widely recognized as a critical “re-formation window” for PCDD/Fs in industrial thermal systems [46]. In this range, heterogeneous pathways may regenerate PCDD/Fs on fly ash surfaces, particularly when oxygen is present together with residual carbon, chlorine sources and catalytic metals such as Cu and Fe. Therefore, while our meta-analysis indicates a net positive removal response under oxygen-limited pyrolysis conditions within the ≤305.9 °C subgroup, engineering-scale implementations should avoid prolonged residence of solids/off-gas within 250–400 °C under possible oxygen ingress. Practical measures include maintaining a sealed/inert atmosphere and minimizing residence time through rapid heating/cooling (quenching) across this window. However, at excessively high temperatures, secondary reactions may occur among small-molecule pyrolysis products. Alternatively, when temperatures exceed 900.0 °C, changes in the physical and chemical properties of fly ash alter its structure, affecting the diffusion and transport of reactants and products within it, reducing removal efficiency [47]. In the pyrolysis time dimension (Figure 4d), dioxin removal efficiency generally increases as residence time extends to an effective window (50–90 min). Duration exceeding 50 min, the wide confidence interval indicates sensitivity to time variations during this phase. Between 50 and 90 min, the removal efficiency reached its maximum increase of approximately 21% compared to the control group. The effect of weight (Figure 4e) on removal efficiency is not significant. Forest plot analysis shows that when the weight ranges from 5 to 50 g, the removal efficiency increases by approximately 60%. However, when the weight is less than or equal to 5 g or greater than or equal to 50 g, the improvement in removal efficiency is not significant. This may occur because excessively low weight leads to uneven distribution within the pyrolysis system, resulting in low heat transfer efficiency. Conversely, high weight restricts weight transfer processes within the system, preventing internal fly ash particles from adequately contacting pyrolysis gases and heat, thereby limiting improvements in removal efficiency [48,49]. Regarding initial concentration (Figure 4f), removal efficiency gradually decreased with increasing concentration. This primarily occurred because at excessively high concentrations, PCDD/Fs molecules rapidly occupied the fly ash surface, hindering subsequent molecules from approaching active sites for pyrolysis reactions, thereby reducing overall removal efficiency [50].

#### 3.2.3. Dioxin Molecular Feature Effects

**Figure 5:**
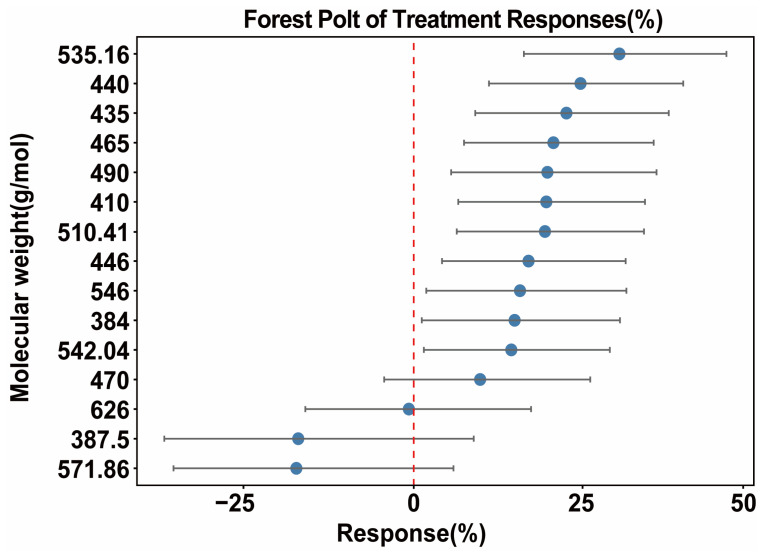
Forest plot of pollutant molecular weights. Blue circles represent the point estimates of the weighted mean *RR* for each subgroup, with their horizontal position indicating the direction and magnitude of the effect. Gray horizontal lines denote the 95% Cl for this effect size, reflecting the precision of the estimate. Red dashed vertical lines mark the null line (*RR* = 0).

The molecular weight of dioxin quantifies its molecular composition and chemical bond structure, directly correlating with key parameters such as molecular size and functional group count. Among dioxins, each additional chlorine atom increases molecular weight by approximately 35.5. This not only reflects the degree of substitution but also influences degradation behavior through intermolecular forces. Therefore, when analyzing PCDD/Fs characteristics, studies focused solely on molecular weight, grouping dioxin isomers by molecular weight category. Dioxins with molecular weights of 384.0 and 387.5 exhibit unique removal efficiency responses (Figure 5). The removal efficiency response for the 384 molecular-weight was at a higher level, showing an approximately 12% increase, with a narrower confidence interval. This indicates relatively high stability and reliability in removal efficiency at this molecular weight. Additionally, compounds with molecular weights of 571.9 and 626.0 warrant particular attention. For the substance with a molecular weight of 571.9, the removal efficiency decreased by approximately 20%, accompanied by a longer confidence interval. This indicates significant uncertainty in the measurement of its removal efficiency. For the substance with a molecular weight of 626.0, the removal efficiency response remained at a low level, showing a decrease of about 2% compared to the control group. These two categories of substances may be difficult to utilize effectively during pyrolysis due to their excessive molecular weight and associated physicochemical properties, thereby reducing removal efficiency. Substances with molecular weights intermediate between high and low molecular weight, such as 440.0 and 460.0, exhibited positive removal efficiency responses. Compared to the 2,3,7,8-PCDD control group, their removal efficiency increased, though the effect was not significant.

Cross-interactions between dioxin molecular properties and operational parameters play an important role in shaping removal behavior, yet this aspect has not been sufficiently emphasized in previous analyses. Dioxin characteristics such as melting point, lg*K*_ow_, and initial concentration influence adsorption, volatilization, and decomposition pathways; however, their effects are highly dependent on the surrounding reaction environment. For example, high temperatures tend to diminish differences in molecular stability, thereby weakening the relative contribution of melting point or hydrophobicity to removal efficiency. In contrast, under low-temperature or short-residence-time conditions, intrinsic molecular properties exert greater influence because the system lacks sufficient energy to override structural differences. Similarly, gas composition and flow rate modulate the influence of molecular properties by altering oxygen availability, diffusion rates, and the formation of reactive intermediates. These interactions indicate that molecular properties do not act independently but interact synergistically or antagonistically with operational parameters, offering a mechanistic explanation for the variability observed in removal efficiency across different conditions.

### 3.3. Key Predictors Identified by Stepwise Regression

Figure S1 presents the results of a stepwise regression analysis conducted to identify factors influencing dioxin removal efficiency during fly ash pyrolysis, with the core objective of screening key variables suitable for SEM analysis. The analysis revealed significant variations in the impact of different factors on dioxin removal efficiency. Several factors exhibited extremely low *p*-values, indicating highly significant correlations with removal efficiency. Among these, flow rate and time were particularly prominent [51]. Data on ash and fixed carbon suggest a synergistic mechanism between inorganic matrix and carbon skeleton, potentially exerting a significant impact on dioxin removal efficiency. The behavior of Cr highlights its unique role in fly ash pyrolysis [52]. Combined with the influence of Cl, it is inferred that the formation of Cr-Cl complexes under high temperatures may alter dioxin speciation, thereby affecting removal efficiency. These factors are crucial for dioxin removal efficiency during fly ash pyrolysis and are highly likely to become key variables in subsequent SEM. However, certain factors showed no statistically significant correlation with dioxin removal efficiency [53]. Volatile matter content showed no apparent correlation with removal efficiency. Following established variable selection criteria, statistically insignificant variables like this can be excluded in subsequent SEM. This approach not only optimizes the model by reducing redundant complexity but also enhances its interpretability and validity, enabling the model to more accurately reflect the factors influencing dioxin removal efficiency during fly ash pyrolysis [54].

### 3.4. Analysis via Structural Equation Modeling

**Figure 6:**
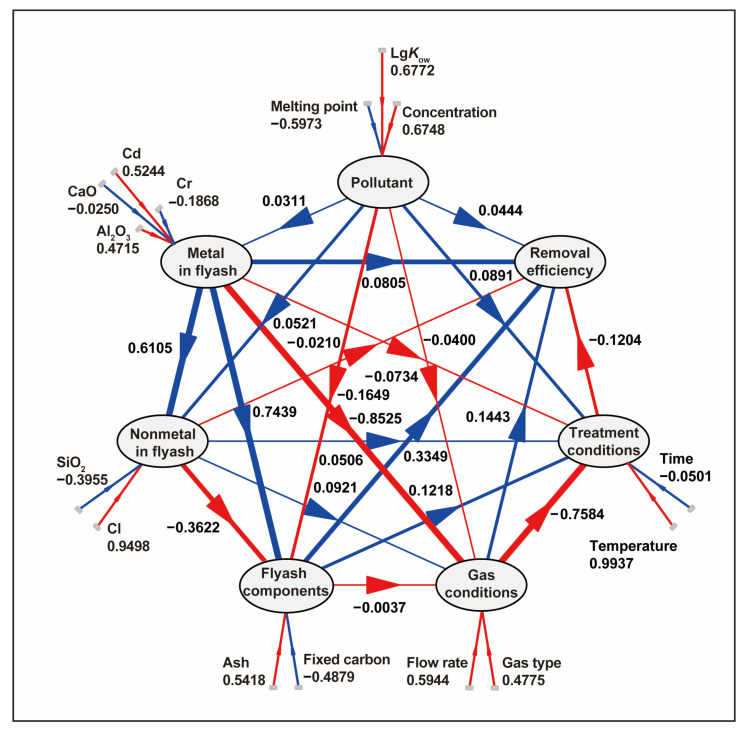
Path diagram of structural equation regression.

**Figure 7:**
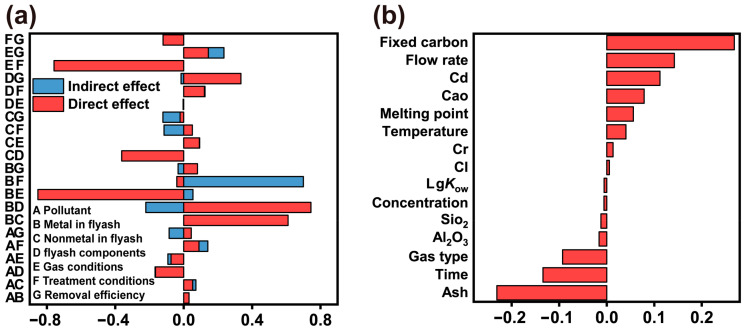
(**a**) Diagram of direct and indirect effects in structural equation regression; (**b**) The effect value of observation indicators on removal efficiency.

During the pyrolysis of fly ash, the removal efficiency of dioxins is regulated by multiple factors, with both direct and indirect interactions existing among them. This study developed a comprehensive SEM describing how metal and nonmetal compositions, fly ash components, gas conditions, and treatment conditions determine the removal efficiency. A correlation analysis of the major oxides (CaO, SiO_2_, Al_2_O_3_) has been added as Figure S2. The results show all absolute Pearson correlation coefficients are ≤0.35 (e.g., CaO vs. SiO_2_: 0.35), significantly below the 0.7 multicollinearity threshold. This confirms no significant collinearity risk, supporting the stability of the SEM model. The outer model evaluation demonstrates that the latent constructs are adequately measured by their indicators. For the fly ash components, fixed carbon (loading = 0.9679) and ash (loading = −0.9740) exhibit exceptionally high loadings, confirming strong construct validity. Metal-related indicators (Cd, Al_2_O_3_) also show strong loadings (>0.8943), while nonmetal indicators (Cl, SiO_2_) indicate a more heterogeneous structure. Temperature dominates the “operating conditions” construct with a loading of −0.9987, suggesting it is the primary driver of this latent variable. Overall, the SEM shows satisfactory indicator reliability and construct validity. In the structural model, fly ash components showed the strongest direct positive effect on removal efficiency (β = 0.3349), followed by gas conditions and metal contents. Conversely, nonmetal composition and treatment conditions had negative direct effects (Figure 6). When considering both direct and indirect pathways, the total effect ranking confirmed fly ash components as the most influential factor (total effect = 0.3194), ahead of gas atmosphere (0.2357). Notably, significant negative total effects were observed for both nonmetal composition and treatment conditions (Figure 7a). At the individual indicator level, the results aligned with the structural model. Fixed carbon was identified as the single strongest positive predictor of removal efficiency (β = 0.2687), whereas its counterpart, ash content, was the strongest negative predictor (β = −0.2313). Other significant positive influences included flow rate and Cd, while time and gas type were major negative predictors (Figure 7b). An integrated mechanistic interpretation synthesizes these findings: High fixed carbon and low ash content enhance fly ash reactivity, directly boosting removal efficiency. A higher flow rate improves gas–solid contact, thereby increasing efficiency. While metals can indirectly facilitate reactions, nonmetallic components adversely affect fly ash stability. Furthermore, longer reaction time decrease removal efficiency, whereas elevated temperature exerts only a mild positive influence. This negative effect reflects the partial (net) contribution of time after controlling for correlated operating variables, suggesting diminishing returns or reduced efficiency under excessively prolonged residence time beyond the effective window.

## 4. Conclusions

This study systematically conducted a meta-analysis to comprehensively evaluate key factors influencing dioxin removal efficiency during fly ash pyrolysis, revealing the complex mechanisms among the physicochemical properties of fly ash, pyrolysis conditions, and dioxin characteristics. Results indicate that different chemical constituents in fly ash exert divergent effects on dioxin removal: Al_2_O_3_, CaO, SiO_2_, and Cl significantly enhance dioxin removal, whereas Fe_2_O_3_ inhibits pyrolysis reactions when its content reaches or exceeds 5.7%. Cd and Cr exhibit a unique bimodal effect: they promote dioxin removal at both low and high concentrations but inhibit removal at moderate concentrations. Regarding pyrolysis conditions, the study found that high temperatures (500–900 °C), extended residence times (50–90 min), elevated flow rates (400 mL/min), and moderate weights (5–50 g) all significantly enhance dioxin removal efficiency. The results of SEM showed that fly ash components are the paramount factor determining removal efficiency, with gas conditions also being highly significant. In contrast, nonmetal composition and prolonged reaction time beyond the optimal window (50–90 min) may impair performance, providing clear quantitative insights for optimizing the process. The innovation of this study lies in achieving a quantitative assessment of the direction and intensity of various influencing factors, while clearly defining the threshold ranges of key parameters. Future research may incorporate machine-learning-based predictive models (e.g., neural networks) to enhance the accuracy and generalizability of pyrolysis performance prediction. The analytical framework developed in this study is also, in principle, transferable to other matrices such as biomass ash and industrial residues, provided that sufficiently standardized and comparable datasets become available. In addition, more comprehensive material-structural characterization, controlled experimental validation of key elemental thresholds, standardized kinetic datasets for mechanistic modeling, and deeper consideration of secondary formation and recombination pathways will further strengthen the connection between statistical inference and the chemistry of pyrolysis.

## Data Availability

The raw data supporting the conclusions of this article will be made available by the authors on request.

## Supplementary Materials

The following supporting information can be downloaded at https://www.mdpi.com/article/10.3390/toxics13121072/s1. Text S1: Literature search criteria; Table S1: Experimental conditions grouping and meta-analysis data; Table S2: Ash properties grouping and meta-analysis data; Table S3: Dioxin properties grouping and meta-analysis data; Table S4: Dioxin contaminants and their corresponding molecular weights; Table S5; Gas types and their relative molecular masses; Table S6: The results of leave-one-study-out sensitivity analysis; Figure S1: Stepwise regression plots. (a) Flow rate; (b) Time; (c) Ash; (d) Fixed carbon; (e) Cd; (f) Cl; (g) Concentration; (h) Cr; (i) Fe_2_O_3_; (j) Boiling point; (k) Al_2_O_3_; (l) Gas type; (m) Henry constant; (n) Lg*K*_ow_; (o) Melting point; (p) SiO_2_; (q) Weight; (r) Volatile matter; (s) CaO; (t) Temperature; Figure S2: Correlation heatmap of oxides in fly ash.

## Abbreviations

The following abbreviations are used in this manuscript:

PCDD/Fs	Polychlorinated dibenzodioxins and polychlorinated dibenzofurans
SEM	Structural Equation Modeling
MSW	Municipal solid waste
MSWI	Municipal solid waste incineration
TEQ	Toxicity equivalent
PRISMA	Systematic reviews and meta-analyses
IQR	Interquartile range
RR	Response ratio

## References

[1] Meena M.D., Dotaniya M.L., Meena B.L., Rai P.K., Antil R.S., Meena H.S., Meena L.K., Dotaniya C.K., Meena V.S., Ghosh A. (2023). Municipal Solid Waste: Opportunities, Challenges and Management Policies in India: A Review. Waste Manag. Bull..

[2] Chicaiza-Ortiz C., Peñafiel-Arcos P., Herrera-Feijoo R.J., Ma W., Logroño W., Tian H., Yuan W. (2024). Waste-to-Energy Technologies for Municipal Solid Waste Management: Bibliometric Review, Life Cycle Assessment, and Energy Potential Case Study. J. Clean. Prod..

[3] Liu B., Wang P., Zhou J., Guo Y., Ma S., Chen W.-Q., Li J., Chang V.W.-C. (2024). Refocusing on Effectiveness over Expansion in Urban Waste–Energy–Carbon Development in China. Nat. Energy.

[4] Teng F., Wang Z., Ren K., Liu S., Ding H. (2024). Analysis of Composition Characteristics and Treatment Techniques of Municipal Solid Waste Incineration Fly Ash in China. J. Environ. Manag..

[5] Yang D., Kow K.-W., Wang W., Meredith W., Zhang G., Mao Y., Xu M. (2024). Co-Treatment of Municipal Solid Waste Incineration Fly Ash and Alumina-/Silica-Containing Waste: A Critical Review. J. Hazard. Mater..

[6] Yue Y., Wen T., Li C., He Y., Fu J., Qu X., Ding J., Zhang H. (2025). Multi-Target Inhibition of Microcystis by Allelochemicals: Linking Oxidative Stress, Photosynthetic Collapse, and Toxin Suppression. J. Hazard. Mater..

[7] Xu D. (2024). Sustainable Solution Selection for Solid Waste Incineration Fly Ash: A Multicriteria Framework Based on Objective Weights and Fusion Ranks. J. Mater. Cycles Waste Manag..

[8] Bouzar B., Mamindy-Pajany Y., Hurel C. (2023). Innovative Reuse of Fly Ashes for Treatment of a Contaminated River Sediment: Synthesis of Layered Double Hydroxides (LDH) and Chemical Performance Assessments. Waste Biomass Valoris..

[9] Zhang Y., Wang L., Chen L., Ma B., Zhang Y., Ni W., Tsang D.C.W. (2021). Treatment of Municipal Solid Waste Incineration Fly Ash: State-of-the-Art Technologies and Future Perspectives. J. Hazard. Mater..

[10] Zhiliang C., Minghui T., Shengyong L., Buekens A., Jiamin D., Qili Q., Jianhua Y. (2019). Mechanochemical Degradation of PCDD/Fs in Fly Ash within Different Milling Systems. Chemosphere.

[11] Duan Y., Liu X., Khalid Z., Jiang X. (2023). Effect of the MgO/SiO2 Ratio on MgO–Silica Binders Solidifying MSWI Fly Ash. Waste Dispos. Sustain. Energy.

[12] Lan T., Meng Y., Ju T., Chen Z., Du Y., Deng Y., Song M., Han S., Jiang J. (2022). Synthesis and Application of Geopolymers from Municipal Waste Incineration Fly Ash (MSWI FA) as Raw Ingredient—A Review. Resour. Conserv. Recycl..

[13] Xie K., Hu H., Xu S., Chen T., Huang Y., Yang Y., Yang F., Yao H. (2020). Fate of Heavy Metals during Molten Salts Thermal Treatment of Municipal Solid Waste Incineration Fly Ashes. Waste Manag..

[14] Jiang X., Zhao Y., Yan J. (2022). Disposal Technology and New Progress for Dioxins and Heavy Metals in Fly Ash from Municipal Solid Waste Incineration: A Critical Review. Environ. Pollut..

[15] Tao H.-C., Lei T., Shi G., Sun X.-N., Wei X.-Y., Zhang L.-J., Wu W.-M. (2014). Removal of Heavy Metals from Fly Ash Leachate Using Combined Bioelectrochemical Systems and Electrolysis. J. Hazard. Mater..

[16] Deng D., Qiao J., Liu M., Kołodyńska D., Zhang M., Dionysiou D.D., Ju Y., Ma J., Chang M. (2019). Detoxification of Municipal Solid Waste Incinerator (MSWI) Fly Ash by Single-Mode Microwave (MW) Irradiation: Addition of Urea on the Degradation of Dioxin and Mechanism. J. Hazard. Mater..

[17] Zia U.U.R., Rashid T.U., Ali M., Awan W.N. (2020). Techno-Economic Assessment of Energy Generation through Municipal Solid Waste: A Case Study for Small/Medium Size Districts in Pakistan. Waste Dispos. Sustain. Energy.

[18] Yang J., Yan M., Li X., Chen T., Lu S., Yan J., Buekens A. (2014). Influence of temperature and atmosphere on polychlorinated dibenzo-p-dioxins and dibenzofurans desorption from waste incineration fly ash. Environ. Technol..

[19] Ying S., Peng Y., Zhang C., Ding J., Zhu Z., Sun S., Zhao X., Lu S. (2025). Influence of Chlorine Removal and CaO Addition on Low-Temperature Pyrolysis of PCDD/Fs in MSWI Fly Ash. J. Environ. Chem. Eng..

[20] Tang Z., Zhang W., Tu Q. (2025). Life Cycle Assessment of Priority Biochemicals: A Review and Meta-Regression Analysis. Resour. Conserv. Recycl..

[21] Liao N., Chen Z., Zhang L., Chen M., Zhang Y., Li J., Wang H. (2024). Study on the Spatiotemporal Distribution of Algal Blooms and Its Influencing Factors in Young Reservoirs Based on Remote Sensing Interpretation. J. Environ. Manag..

[22] Fraszczyk M., Kaczmarek-Majer K., Hryniewicz O., Skotak K., Degorska A. Expert-in-the-Loop Stepwise Regression and Its Application in Air Pollution Modeling. Proceedings of the 2022 IEEE 11th International Conference on Intelligent Systems (IS).

[23] Zhou S.-Y., Huang A.-C., Wu J., Wang Y., Wang L.-S., Zhai J., Xing Z.-X., Jiang J.-C., Huang C.-F. (2022). Establishment and Assessment of Urban Meteorological Disaster Emergency Response Capability Based on Modeling Methods. Int. J. Disaster Risk Reduct..

[24] Baquero M., Cifrian E., Pérez-Gandarillas L., Andrés A. (2021). Methodology proposed for estimating biowaste generation using municipal rurality indexes. Waste Biomass Valoris..

[25] Lou P., Wu T., Yin G., Chen J., Zhu X., Wu X., Li R., Yang S. (2024). A Novel Framework for Multiple Thermokarst Hazards Risk Assessment and Controlling Environmental Factors Analysis on the Qinghai-Tibet Plateau. CATENA.

[26] Van Gerwen M., Vasan V., Genden E., Saul S.R. (2023). Human 2,3,7,8-Tetrachlorodibenzo-p-Dioxin Exposure and Thyroid Cancer Risk. Toxicology.

[27] Li Y., Sidikjan N., Huang L., Chen Y., Zhang Y., Li Y., Yang J., Shen G., Liu M., Huang Y. (2024). Multi-Media Environmental Fate of Polychlorinated Dibenzo-p-Dioxins and Dibenzofurans in China: A Systematic Review of Emissions, Presence, Transport Modeling and Health Risks. Environ. Pollut..

[28] Crowther M., Lim W., Crowther M.A. (2010). Systematic Review and Meta-Analysis Methodology. Blood.

[29] Harrer M., Cuijpers P., Furukawa T., Ebert D. (2021). Doing Meta-Analysis with R-a Hands-on Guide.

[30] Schwarzer G., Carpenter J.R., Rücker G. (2015). Meta-analysis with binary outcomes. Meta-Analysis with R.

[31] Bakbergenuly I., Hoaglin D.C., Kulinskaya E. (2020). Estimation in meta-analyses of response ratios. BMC Med. Res. Methodol..

[32] Erni-Cassola G., Zadjelovic V., Gibson M.I., Christie-Oleza J.A. (2019). Distribution of Plastic Polymer Types in the Marine Environment; a Meta-Analysis. J. Hazard. Mater..

[33] Knox L., Karantzas G.C., Romano D., Feeney J.A., Simpson J.A. (2022). One year on: What we have learned about the psychological effects of COVID-19 social restrictions: A meta-analysis. Curr. Opin. Psychol..

[34] Goyal R., Singha P., Singh S.K. (2024). Spectroscopic Food Adulteration Detection Using Machine Learning: Current Challenges and Future Prospects. Trends Food Sci. Technol..

[35] Migliorini M., Fierri I., Zoccatelli G., Chignola R. (2024). Chemdeg, an R Package for the Analysis of Foods Isothermal Degradation Kinetics. J. Food Eng..

[36] Vikrant S., Ramachandran M., Sathiyaraj C., Vimala S. (2021). A Review on Structural Equation Modeling and Its Classification. REST J. Emerg. Trends Model. Manuf..

[37] Hidayat R., Wulandari P. (2022). Structural Equation Modelling (SEM) in Research: Narrative Literature Review. Open Access Indones. J. Soc. Sci..

[38] Zhang D., Liu Y., Shao Y., Ma C., Sun H., Wang B., Zhu L. (2023). Mineral Evolution Mechanism of Zinc-Slag during High-Temperature Reactive Process. Mater. Today Commun..

[39] Anakhu E.A., Ameh V.I., Modekwe H.U., Ayeleru O.O., Ramatsa I.M. (2023). Remediation of Cadmium and Chromium Using Modified Vitex Doniana Waste Plant Seed’s Biochar in Quarry Site Surface Water. Environ. Funct. Mater..

[40] Kabir E., Kim K.-H., Kwon E.E. (2023). Biochar as a Tool for the Improvement of Soil and Environment. Front. Environ. Sci..

[41] Mao L., Deng N., Liu L., Cui H., Zhang W. (2016). Effects of Al_2_O_3_, Fe_2_O_3_, and SiO_2_ on Cr(VI) Formation during Heating of Solid Waste Containing Cr(III). Chem. Eng. J..

[42] Yu S., Zhang C., Ma L., Tan P., Fang Q., Chen G. (2021). Deep Insight into the Effect of NaCl/HCl/SO_2_/CO_2_ in Simulated Flue Gas on Gas-Phase Arsenic Adsorption over Mineral Oxide Sorbents. J. Hazard. Mater..

[43] Wang L., Zhang F., Chen J. (2001). Carbonyl Sulfide Derived from Catalytic Oxidation of Carbon Disulfide over Atmospheric Particles. Environ. Sci. Technol..

[44] Pivato A., Gohar H., Antille D., Schievano A., Beggio G., Reichardt P., Maria F., Peng W., Castegnaro S., Lavagnolo M. (2024). Air-Polluting Emissions from Pyrolysis Plants: A Systematic Mapping. Environments.

[45] Ahmaruzzaman M. (2009). Role of Fly Ash in the Removal of Organic Pollutants from Wastewater. Energy Fuels.

[46] Duan H., Li J., Liu Y., Yamazaki N., Jiang W. (2011). Characterization and Inventory of PCDD/Fs and PBDD/Fs Emissions from the Incineration of Waste Printed Circuit Board. Environ. Sci. Technol..

[47] Acharya B.S., Dodla S., Wang J.J., Pavuluri K., Darapuneni M., Dattamudi S., Maharjan B., Kharel G. (2024). Biochar Impacts on Soil Water Dynamics: Knowns, Unknowns, and Research Directions. Biochar.

[48] Setya Wardhana B., Hanum F.F., Mufrodi Z., Jamilatun S. (2024). Review: Effect of Material Characteristics, and Process Conditions in Reducing Gaseous Pollutants Using Fly Ash (FA)-Based Adsorbent. Sains Nat. J. Biol. Chem..

[49] Juntarasakul O., Roongcharoen P., Tabelin C.B., Phengsaart T. (2024). Municipal Solid Waste Incineration (MSWI) Fly Ash as a Potential Adsorbent for Phosphate Removal. Sustainability.

[50] Costa S.I.G., Ferreira F.L., Weschenfelder S.E., Fuck J.V.R., da Cunha M.d.F.R., Marinho B.A., Mazur L.P., da Silva A., de Souza S.M.A., Guelli U. (2023). Towards the Removal of Soluble Organic Compounds Present in Oilfield Produced Water by Advanced Oxidation Processes: Critical Review and Future Directions. Process Saf. Environ. Prot..

[51] Chen W., Li P., Zhang S., Chen Y. (2025). Mass Distribution of Organic Carbon, S-Containing Compounds and Heavy Metals during Flotation of Municipal Solid Waste Incineration Fly Ash. Recycling.

[52] Lin X., Guo Y., Tang B., Fu P., Li H., Zhang J., Li P. (2024). Fast Co-Pyrolysis of Paper Mill Sludge and Corn Stover: Relationships between Parameters, Product Distributions, and Synergistic Interactions. Ind. Crops Prod..

[53] Deng H., Tu Y., Wang H., Wang Z., Li Y., Chai L., Zhang W., Lin Z. (2022). Environmental Behavior, Human Health Effect, and Pollution Control of Heavy Metal(Loid)s toward Full Life Cycle Processes. Eco-Environ. Health.

[54] Cheng Z., Liu Y., Wu J., Guo X., Chen W., Gao Y. (2023). Graphene Oxide-Coated Fly Ash for High Performance and Low-Carbon Cementitious Composites. J. Mater. Res. Technol..

