# Supplementary material for: Quantifying Influencing Factors of Dioxin Removal in Fly Ash Pyrolysis Through Meta-Analysis and Structural Equation Modeling
Source: Toxics. 2025 Dec 12;13(12):1072. doi: 10.3390/toxics13121072 (PMC12737346; doi:10.3390/toxics13121072)
Supplement: Supplementary file 1 [file toxics-13-01072-s001.zip › toxics-4016893-supplementary.pdf]

Supplementary Material

Quantifying Influencing Factors of Dioxin Removal in Fly Ash Pyrolysis Through Meta-Analysis and Structural Equation Modeling

Tao He [1,2], Shihan Tan [1,2], Qi Su [3], Feifei Chen [4], Chenlei Xie [1,2], Yuchi Zhong [4], Shuai Zhang [1,2,*] and Jiafeng Ding [1,2]

[1] School of Engineering, Hangzhou Normal University, Hangzhou 310018, China
[2] Zhejiang Provincial Key Laboratory of Wetland Intelligent Monitoring and Ecological Restoration, Hangzhou 311121, China
[3] Eco-Environmental Science Research and Design Institute of Zhejiang Province, Hangzhou 311121, China
[4] Hangzhou Fuyang Huilong Environmental Protection Technology Co., Ltd., Hangzhou 330183, China
* Correspondence: zs@hznu.edu.cn

Legend of Contents

Text S1. Literature search criteria.

Table S1. Experimental conditions grouping and meta-analysis data.

Table S2. Ash properties grouping and meta-analysis data.

Table S3. Dioxin properties grouping and meta-analysis data.

Table S4. Dioxin contaminants and their corresponding molecular weights.

Table S5. Gas types and their relative molecular masses.

Table S6. The results of leave-one-study-out sensitivity analysis.

Figure S1. Stepwise regression plots. (a) Flow rate; (b) Time; (c) Ash; (d) Fixed carbon; (e) Cd; (f) Cl; (g) Concentration; (h) Cr; (i) Fe_2O_3; (j) Boiling point; (k) Al_2O_3; (l) Gas type; (m) Henry constant; (n) LgK_{ow}; (o) Melting point; (p) SiO_2; (q) Weight; (r) Volatile matter; (s) CaO; (t) Temperature.

Figure S2. Correlation heatmap of oxides in fly ash.

Text S1. Literature search criteria.

In this study, the literature was retrieved using topic terms combined with Boolean operators through the advanced search tools of Web of Science and Google Scholar. The Web of Science topic search was conducted using the following query: TS=(“fly ash”) AND TS=(“pyrolysis” OR “thermal treatment” OR “thermal process” OR “thermal degradation”) AND TS=(“organic pollutant*” OR “persistent organic pollutant*” OR “POPs” OR “dioxin*” OR contaminant*) AND TS=(“removal” OR “reduction” OR “destruction” OR “degradation” OR “elimination” OR “treatment”), where TS stands for topic term search; “OR” and “AND” were used to combine synonym sets and concept blocks into one integrated query expression. The publication date range was limited to January 2010–June 2025, the document type was restricted to article, and the language was restricted to English. The same keyword combinations and Boolean logic were applied in Google Scholar to complement the retrieval.

Table S1. Experimental conditions grouping and meta-analysis data.

Variables	Groups	n	p	I^2 (%)
	≤5	1721		
Weight (g)	5-50	2266	<0.00001	94
	≥50	41		
	≤305.9	1316		
Temperature (℃)	305.9-900	1408	<0.0001	90
	≥900	1289		
	≤50	373		
Time (min)	50-90	3342	0.74	0
	≥90	313		
	≤200	446		
Flow rate (min)	200-400	1253	0.05	66
	≥400	2329		
	≤28	1960		
Gas type	28-29	165	<0.00001	94
	≥29	1903		

I^2 (%) indicates the degree of heterogeneity: 25%=low, 50%=moderate, 75%=high.

Table S2. Ash properties grouping and meta-analysis data.

Variables	Groups	n	p	I^2 (%)
	≤7.74	1678		
Fixed carbon	7.74-19.55	1818	0.06	65
	≥19.55	532		
	≤18.66	1322		
SiO_2	18.66-22.58	1227	0.97	0
	≥22.58	1479		
	≤2.22	1345		
Fe_2O_3	2.22-5.74	2459	0.004	82
	≥5.74	224		
	≤0.0644	1434		
Cr	0.0644-0.586	1314	<0.0001	90
	≥0.586	1280		
	≤7.81	1579		
Cl	7.81-14.4	1066	0.05	66
	≥14.4	1241		
	≤0.014	1722		
Cd	0.014-0.67	942	<0.00001	93
	≥0.67	1339		
	≤22.06	1104		
CaO	22.06-25.93	1697	0.74	0
	≥25.93	1227		
	≤5.14	1787		
Al_2O_3	5.14-13	1958	0.36	2
	≥13	283		

I^2 (%) indicates the degree of heterogeneity: 25%=low, 50%=moderate, 75%=high.

Table S3. Dioxin properties grouping and meta-analysis data.

Variables	Groups	n	p	I^2 (%)
	≤199.494	1343		
Concentration (pg/g)	199.494-1337.196	1353	＜0.00001	95
	≥1337.196	1332		

I^2 (%) indicates the degree of heterogeneity: 25%=low, 50%=moderate, 75%=high.

Table S4. Dioxin contaminants and their corresponding molecular weights.

Abbreviation	Full name	Molecular weight
1,3,7,9-TCDD	1,3,7,9-Tetrachlorodibenzo-p-dioxin	446
1,3,6,9-TCDD	1,3,6,9-Tetrachlorodibenzo-p-dioxin	446
1,2,4,7-TCDD	1,2,4,7-Tetrachlorodibenzo-p-dioxin	446
1,2,4,8-TCDD	1,2,4,8-Tetrachlorodibenzo-p-dioxin	446
1,2,4,6-TCDD	1,2,4,6-Tetrachlorodibenzo-p-dioxin	446
1,2,4,9-TCDD	1,2,4,9-Tetrachlorodibenzo-p-dioxin	446
1,3,7,8-TCDD	1,3,7,8-Tetrachlorodibenzo-p-dioxin	446
1,4,6,9-TCDD	1,4,6,9-Tetrachlorodibenzo-p-dioxin	446
1,2,6,8-TCDD	1,2,6,8-Tetrachlorodibenzo-p-dioxin	446
1,4,7,8-TCDD	1,4,7,8-Tetrachlorodibenzo-p-dioxin	446
1,2,7,9-TCDD	1,2,7,9-Tetrachlorodibenzo-p-dioxin	446
1,2,3,4-TCDD	1,2,3,4-Tetrachlorodibenzo-p-dioxin	446
1,2,6,9-TCDD	1,2,6,9-Tetrachlorodibenzo-p-dioxin	446
1,2,3,6-TCDD	1,2,3,6-Tetrachlorodibenzo-p-dioxin	446
1,2,3,7-TCDD	1,2,3,7-Tetrachlorodibenzo-p-dioxin	446
1,2,3,8-TCDD	1,2,3,8-Tetrachlorodibenzo-p-dioxin	446
2,3,7,8-TCDD	2,3,7,8-Tetrachlorodibenzo-p-dioxin	446
1,2,3,9-TCDD	1,2,3,9-Tetrachlorodibenzo-p-dioxin	446
1,2,7,8-TCDD	1,2,7,8-Tetrachlorodibenzo-p-dioxin	446
1,2,6,7-TCDD	1,2,6,7-Tetrachlorodibenzo-p-dioxin	446
1,2,8,9-TCDD	1,2,8,9-Tetrachlorodibenzo-p-dioxin	446
1,3,6,8-TCDD	1,3,6,8-Tetrachlorodibenzo-p-dioxin	446
1,2,4,6,8-PeCDD	1,2,4,6,8-Pentachlorodibenzo-p-dioxin	465
1,2,4,7,9-PeCDD	1,2,4,7,9-Pentachlorodibenzo-p-dioxin	465
1,2,4,6,9-PeCDD	1,2,4,6,9-Pentachlorodibenzo-p-dioxin	465
1,2,3,6,8-PeCDD	1,2,3,6,8-Pentachlorodibenzo-p-dioxin	465
1,2,4,7,8-PeCDD	1,2,4,7,8-Pentachlorodibenzo-p-dioxin	465

Table S4. Dioxin contaminants and their corresponding molecular weights.

Abbreviation	Full name	Molecular weight
1,2,3,7,9-PeCDD	1,2,3,7,9-Pentachlorodibenzo-p-dioxin	465
1,2,3,6,9-PeCDD	1,2,3,6,9-Pentachlorodibenzo-p-dioxin	465
1,2,4,6,7-PeCDD	1,2,4,6,7-Pentachlorodibenzo-p-dioxin	465
1,2,4,8,9-PeCDD	1,2,4,8,9-Pentachlorodibenzo-p-dioxin	465
1,2,3,4,7-PeCDD	1,2,3,4,7-Pentachlorodibenzo-p-dioxin	465
1,2,3,4,6-PeCDD	1,2,3,4,6-Pentachlorodibenzo-p-dioxin	465
1,2,3,7,8-PeCDD	1,2,3,7,8-Pentachlorodibenzo-p-dioxin	465
1,2,3,6,7-PeCDD	1,2,3,6,7-Pentachlorodibenzo-p-dioxin	465
1,2,3,8,9-PeCDD	1,2,3,8,9-Pentachlorodibenzo-p-dioxin	465
1,2,4,6,7,9-HxCDD	1,2,4,6,7,9-Hexachlorodibenzo-p-dioxin	435
1,2,4,6,8,9-HxCDD	1,2,4,6,8,9-Hexachlorodibenzo-p-dioxin	435
1,2,3,4,6,8-HxCDD	1,2,3,4,6,8-Hexachlorodibenzo-p-dioxin	435
1,2,3,6,7,9-HxCDD	1,2,3,6,7,9-Hexachlorodibenzo-p-dioxin	435
1,2,3,6,8,9-HxCDD	1,2,3,6,8,9-Hexachlorodibenzo-p-dioxin	435
1,2,3,4,6,9-HxCDD	1,2,3,4,6,9-Hexachlorodibenzo-p-dioxin	435
1,2,3,4,7,8-HxCDD	1,2,3,4,7,8-Hexachlorodibenzo-p-dioxin	435
1,2,3,6,7,8-HxCDD	1,2,3,6,7,8-Hexachlorodibenzo-p-dioxin	546
1,2,3,4,6,7-HxCDD	1,2,3,4,6,7-Hexachlorodibenzo-p-dioxin	435
1,2,3,7,8,9-HxCDD	1,2,3,7,8,9-Hexachlorodibenzo-p-dioxin	470
1,2,3,4,6,7,9-HpCDD	1,2,3,4,6,7,9-Heptachlorodibenzo-p-dioxin	490
1,2,3,4,6,7,8-HpCDD	1,2,3,4,6,7,8-Heptachlorodibenzo-p-dioxin	490
1,2,3,4,6,7,8,9-OCDD	1,2,3,4,6,7,8,9-Octachlorodibenzo-p-dioxin	535.16
1,3,6,8-TCDF	1,3,6,8-Tetrachlorodibenzofuran	410
1,4,6,8-TCDF	1,4,6,8-Tetrachlorodibenzofuran	410

Table S4. Dioxin contaminants and their corresponding molecular weights.

Abbreviation	Full name	Molecular weight
2,4,6,8-TCDF	2,4,6,8-Tetrachlorodibenzofuran	410
1,2,4,7-TCDF	1,2,4,7-Tetrachlorodibenzofuran	410
1,3,4,7-TCDF	1,3,4,7-Tetrachlorodibenzofuran	410
1,3,7,8-TCDF	1,3,7,8-Tetrachlorodibenzofuran	410
1,3,4,6-TCDF	1,3,4,6-Tetrachlorodibenzofuran	410
1,2,4,6-TCDF	1,2,4,6-Tetrachlorodibenzofuran	410
1,3,6,7-TCDF	1,3,6,7-Tetrachlorodibenzofuran	410
1,3,4,8-TCDF	1,3,4,8-Tetrachlorodibenzofuran	410
1,3,7,9-TCDF	1,3,7,9-Tetrachlorodibenzofuran	410
1,2,4,8-TCDF	1,2,4,8-Tetrachlorodibenzofuran	410
1,2,6,8-TCDF	1,2,6,8-Tetrachlorodibenzofuran	410
1,4,6,7-TCDF	1,4,6,7-Tetrachlorodibenzofuran	410
1,4,7,8-TCDF	1,4,7,8-Tetrachlorodibenzofuran	410
1,3,6,9-TCDF	1,3,6,9-Tetrachlorodibenzofuran	410
1,2,3,7-TCDF	1,2,3,7-Tetrachlorodibenzofuran	410
1,2,3,8-TCDF	1,2,3,8-Tetrachlorodibenzofuran	410
1,2,3,6-TCDF	1,2,3,6-Tetrachlorodibenzofuran	410
1,4,6,9-TCDF	1,4,6,9-Tetrachlorodibenzofuran	410
1,6,7,8-TCDF	1,6,7,8-Tetrachlorodibenzofuran	410
1,2,3,4-TCDF	1,2,3,4-Tetrachlorodibenzofuran	410
2,3,6,8-TCDF	2,3,6,8-Tetrachlorodibenzofuran	410
2,4,6,7-TCDF	2,4,6,7-Tetrachlorodibenzofuran	410
1,2,7,8-TCDF	1,2,7,8-Tetrachlorodibenzofuran	410
1,3,4,9-TCDF	1,3,4,9-Tetrachlorodibenzofuran	410
1,2,6,7-TCDF	1,2,6,7-Tetrachlorodibenzofuran	410
2,3,4,8-TCDF	2,3,4,8-Tetrachlorodibenzofuran	410
2,3,7,8-TCDF	2,3,7,8-Tetrachlorodibenzofuran	410

Table S4. Dioxin contaminants and their corresponding molecular weights.

Abbreviation	Full name	Molecular weight
2,3,4,7-TCDF	2,3,4,7-Tetrachlorodibenzofuran	410
1,2,7,9-TCDF	1,2,7,9-Tetrachlorodibenzofuran	410
2,3,4,6-TCDF	2,3,4,6-Tetrachlorodibenzofuran	410
1,2,4,9-TCDF	1,2,4,9-Tetrachlorodibenzofuran	410
2,3,6,7-TCDF	2,3,6,7-Tetrachlorodibenzofuran	410
3,4,6,7-TCDF	3,4,6,7-Tetrachlorodibenzofuran	410
1,2,6,9-TCDF	1,2,6,9-Tetrachlorodibenzofuran	410
1,2,3,9-TCDF	1,2,3,9-Tetrachlorodibenzofuran	410
1,2,8,9-TCDF	1,2,8,9-Tetrachlorodibenzofuran	410
1,2,4,6,7-PeCDF	1,2,4,6,7-Pentachlorodibenzofuran	440
1,2,3,6,8-PeCDF	1,2,3,6,8-Pentachlorodibenzofuran	440
1,3,4,7,8-PeCDF	1,3,4,7,8-Pentachlorodibenzofuran	440
1,2,4,7,8-PeCDF	1,2,4,7,8-Pentachlorodibenzofuran	440
1,3,4,7,9-PeCDF	1,3,4,7,9-Pentachlorodibenzofuran	440
1,4,6,7,8-PeCDF	1,4,6,7,8-Pentachlorodibenzofuran	440
1,2,4,7,9-PeCDF	1,2,4,7,9-Pentachlorodibenzofuran	440
1,3,4,6,9-PeCDF	1,3,4,6,9-Pentachlorodibenzofuran	440
2,3,4,6,8-PeCDF	2,3,4,6,8-Pentachlorodibenzofuran	440
1,2,4,6,9-PeCDF	1,2,4,6,9-Pentachlorodibenzofuran	440
1,2,3,4,7-PeCDF	1,2,3,4,7-Pentachlorodibenzofuran	440
1,2,3,4,6-PeCDF	1,2,3,4,6-Pentachlorodibenzofuran	440
1,2,3,4,8-PeCDF	1,2,3,4,8-Pentachlorodibenzofuran	440
1,2,3,7,8-PeCDF	1,2,3,7,8-Pentachlorodibenzofuran	440
1,2,3,6,7-PeCDF	1,2,3,6,7-Pentachlorodibenzofuran	440
1,2,6,7,8-PeCDF	1,2,6,7,8-Pentachlorodibenzofuran	440
1,2,3,7,9-PeCDF	1,2,3,7,9-Pentachlorodibenzofuran	440
2,3,4,7,8-PeCDF	2,3,4,7,8-Pentachlorodibenzofuran	384

Table S4. Dioxin contaminants and their corresponding molecular weights.

Abbreviation	Full name	Molecular weight
1,2,4,8,9-PeCDF	1,2,4,8,9-Pentachlorodibenzofuran	440
1,2,6,7,9-PeCDF	1,2,6,7,9-Pentachlorodibenzofuran	440
1,2,3,6,9-PeCDF	1,2,3,6,9-Pentachlorodibenzofuran	440
2,3,4,6,7-PeCDF	2,3,4,6,7-Pentachlorodibenzofuran	440
1,2,3,4,9-PeCDF	1,2,3,4,9-Pentachlorodibenzofuran	440
1,2,3,8,9-PeCDF	1,2,3,8,9-Pentachlorodibenzofuran	440
1,2,3,4,6,8-HxCDF	1,2,3,4,6,8-Hexachlorodibenzofuran	510.41
1,3,4,6,7,8-HxCDF	1,3,4,6,7,8-Hexachlorodibenzofuran	510.41
1,2,4,6,7,8-HxCDF	1,2,4,6,7,8-Hexachlorodibenzofuran	510.41
1,3,4,6,7,9-HxCDF	1,3,4,6,7,9-Hexachlorodibenzofuran	510.41
1,2,4,6,7,9-HxCDF	1,2,4,6,7,9-Hexachlorodibenzofuran	510.41
1,2,4,6,8,9-HxCDF	1,2,4,6,8,9-Hexachlorodibenzofuran	510.41
1,2,3,4,6,7-HxCDF	1,2,3,4,6,7-Hexachlorodibenzofuran	510.41
1,2,3,4,7,8-HxCDF	1,2,3,4,7,8-Hexachlorodibenzofuran	510.41
1,2,3,6,7,8-HxCDF	1,2,3,6,7,8-Hexachlorodibenzofuran	510.41
1,2,3,4,7,9-HxCDF	1,2,3,4,7,9-Hexachlorodibenzofuran	510.41
1,2,3,4,6,9-HxCDF	1,2,3,4,6,9-Hexachlorodibenzofuran	510.41
1,2,3,6,7,9-HxCDF	1,2,3,6,7,9-Hexachlorodibenzofuran	510.41
2,3,4,6,7,8-HxCDF	2,3,4,6,7,8-Hexachlorodibenzofuran	510.41
1,2,3,6,8,9-HxCDF	1,2,3,6,8,9-Hexachlorodibenzofuran	510.41
1,2,3,7,8,9-HxCDF	1,2,3,7,8,9-Hexachlorodibenzofuran	510.41
1,2,3,4,8,9-HxCDF	1,2,3,4,8,9-Hexachlorodibenzofuran	510.41
1,2,3,4,6,7,8-HpCDF	1,2,3,4,6,7,8-Heptachlorodibenzofuran	542.04
1,2,3,4,6,7,9-HpCDF	1,2,3,4,6,7,9-Heptachlorodibenzofuran	542.04
1,2,3,4,6,8,9-HpCDF	1,2,3,4,6,8,9-Heptachlorodibenzofuran	542.04
1,2,3,4,7,8,9-HpCDF	1,2,3,4,7,8,9-Heptachlorodibenzofuran	542.04
OCDD	Octachlorodibenzo-p-dioxin	626

Table S4. Dioxin contaminants and their corresponding molecular weights.

Abbreviation	Full name	Molecular weight
OCDF	Octachlorodibenzofuran	510
1,2,3,4,6,7,8,9-OCDD	1,2,3,4,6,7,8,9-Octachlorodibenzo-p-dioxin	535.16
1,2,3,4,6,7,8,9-OCDF	1,2,3,4,6,7,8,9-Octachlorodibenzofuran	571.86

Table S5. Gas types and their relative molecular masses.

Relative molecular mass	Gas type
28	N_2
28.04	99%N_2 、1%O_2
28.4	90%N_2 、10%O_2
28.84	79%N_2 、21%O_2
29	Air
30	50%N_2 、50%O_2
30.92	27%N_2 、73%O_2

Table S6. The results of leave-one-study-out sensitivity analysis.

study	yi.intrcpt	study	yi.intrcpt
Molecular weight 384	89.63	Cl(%)≥14.4	89.63
Molecular weight 387.5	90.09	Cd(%)≤0.014	89.60
Molecular weight 410	89.56	Cd(%)0.014-0.67	89.64
Molecular weight 435	89.53	Cd(%)>0.67	89.49
Molecular weight 440	89.49	CaO(%)≤22.06	89.72
Molecular weight 446	89.60	CaO(%)22.06-25.93	89.42
Molecular weight 465	89.55	CaO(%)>25.93	89.64
Molecular weight 470	89.69	Al_2O_3(%)≤5.14	89.57
Molecular weight 490	89.57	Al_2O_3(%)5.14-13	89.54
Molecular weight 510.41	89.57	Al_2O_3(%)≥13	89.79
Molecular weight 535.16	89.39	Weight(g)≤5	89.49
Molecular weight 542.04	89.63	Weight(g)5-50	89.64
Molecular weight 546	89.62	Weight(g)50	89.39
Molecular weight 571.86	90.20	Temperature(°C)≤305.9	89.60
Molecular weight 626	89.83	Temperature(°C)305.9-900	89.49
Fixed carbon(%)≤7.74	89.74	Temperature(°C)>900	89.63
Fixed carbon(%)7.74-19.55	89.42	Time(min)≤50	89.61
Fixed carbon(%)>19.55	89.54	Time(min)50-90	89.56
SiO_2(%)≤18.66	89.49	Time(min)>90	89.61
SiO_2(%)18.66-22.58	89.41	Flow rate(ml/min)≤200	89.93
SiO_2(%)>22.58	89.66	Flow rate(ml/min)200-400	89.42
Fe_2O_3(%)≤2.22	89.69	Flow rate(ml/min)>400	89.57
Fe_2O_3(%)2.22-5.74	89.51	Gas type(g/mol)≤28	89.51
Fe_2O_3(%)>5.74	89.57	Gas type(g/mol)28-29	89.65
Cr(%)≤0.0644	89.47	Gas type(g/mol)>29	89.63
Cr(%)0.0644-0.586	89.71	Concentration(pg/g)≤199.494	89.51
Cr (%)>0.586	89.55	Concentration(pg/g)199.494-1337.196	89.64
Cl(%)≤7.81	89.56	Concentration(pg/g)>1337.196	89.56
Cl(%) 7.81-14.4	89.49		

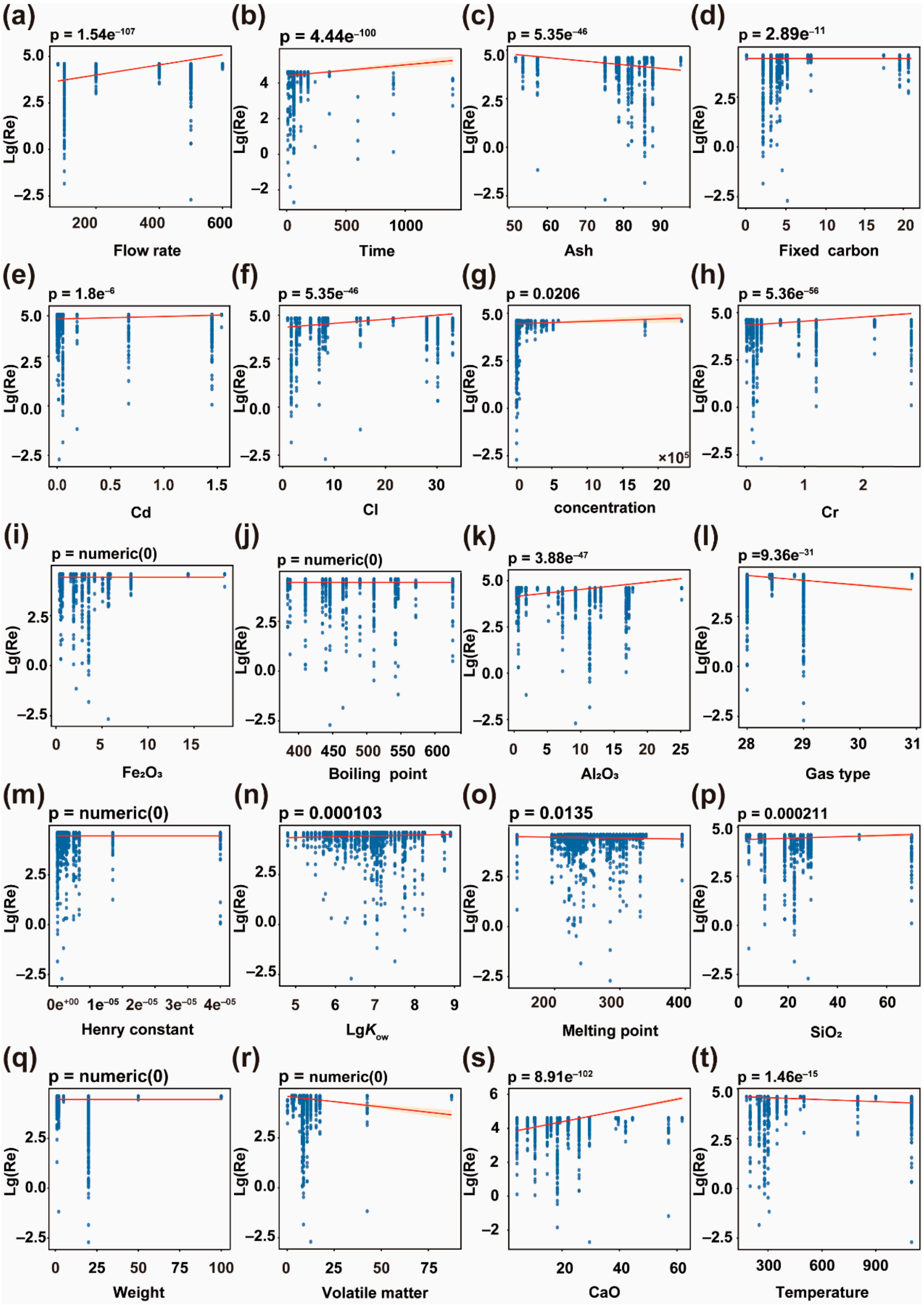

Figure S1. Stepwise regression plots. (a) Flow rate; (b) Time; (c) Ash; (d) Fixed carbon; (e) Cd; (f) Cl; (g) Concentration; (h) Cr; (i) Fe_2O_3; (j) Boiling point; (k) Al_2O_3; (l) Gas type; (m) Henry constant; (n) LgK_{ow}; (o) Melting point; (p) SiO_2; (q) Weight; (r) Volatile matter; (s) CaO; (t) Temperature.

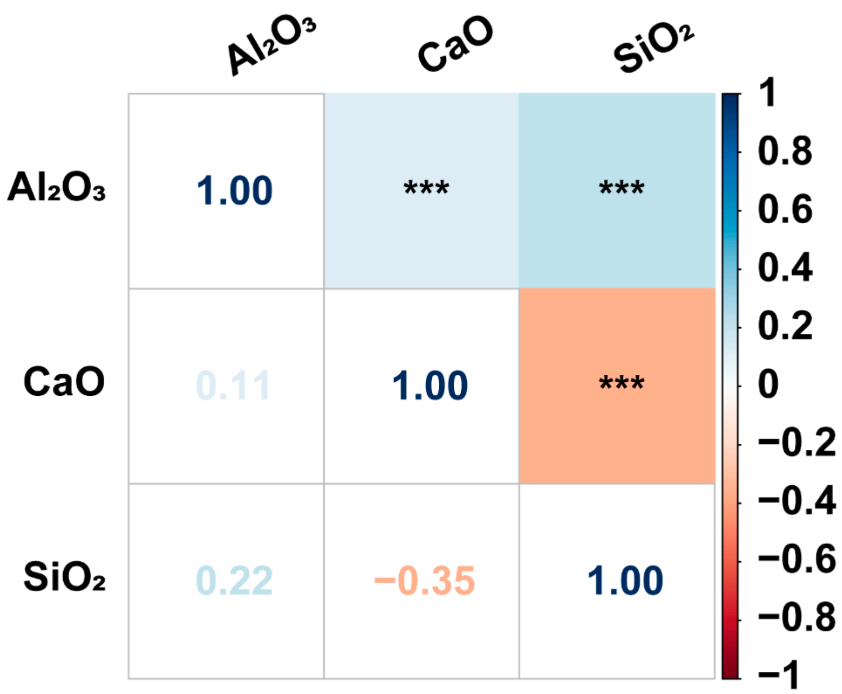

Figure S2. Correlation heatmap of oxides in fly ash.
